# Contribution of Sex‐Biased Expressed Genes in Osteoarthritis

**DOI:** 10.1002/smmd.70019

**Published:** 2025-10-06

**Authors:** Cuicui Wang, Meng Shi, Tiandao Li, Shuang Liu, Liang Fang, Ming Xu, Bo Zhang, Jie Shen

**Affiliations:** ^1^ Department of Orthopaedic Surgery School of Medicine Washington University St. Louis Missouri USA; ^2^ Department of Developmental Biology Center of Regenerative Medicine Washington University St. Louis Missouri USA; ^3^ Masonic Institute on the Biology of Aging and Metabolism University of Minnesota Minneapolis Minnesota USA; ^4^ Department of Chemistry Molecular Biology and Biophysics University of Minnesota Minneapolis Minnesota USA

**Keywords:** cartilage, hormone, osteoarthritis, sex, transcriptomics, transposable element

## Abstract

Human osteoarthritis (OA) displays sex‐specific patterns in its clinical presentation. Key features of the disease—such as prevalence, age of onset, progression, and response to treatment—vary between males and females. These differences have been associated with sex hormones, as well as anatomical, biomechanical, and behavioral distinctions between the sexes. However, the underlying mechanisms driving these sex‐specific disparities in OA pathogenesis remain largely unknown. In this study, we analyzed transcriptomic data from human knee articular cartilage to investigate sex‐specific gene expression in articular chondrocytes. We identified genes that are uniquely or predominantly expressed in either males or females in healthy cartilage. Notably, many of these sex‐biased genes were significantly dysregulated in osteoarthritic cartilage, particularly those with higher expression in females. Furthermore, female‐specific OA genes may exert protective effects on cartilage degeneration, whereas male‐specific OA genes could impair cartilage homeostasis. Our findings provide insights into the genetic regulation of OA and highlight the influence of sex on its molecular pathology.

## Key Points

- Sex‐biased effects on chondrocyte transcriptome were identified in normal articular cartilage.
- Sex‐specific gene profiles were involved in distinct OA biological processes.
- A potential hormone‐responsive regulatory effect was observed in OA pathogenesis.

## 1 Introduction

Osteoarthritis (OA), the most common form of arthritis in developed countries, is a chronic degenerative and disabling joint disorder that affects all joint tissues. With rising obesity rates and increasing life expectancy, the prevalence of OA is expected to grow substantially [1]. Currently, there are no effective disease‐modifying treatments for, partly due to a lack of comprehensive understanding of the pathological mechanisms underlying the initiation and progression of the disease. Although several risk factors such as aging, obesity, joint injury, and genetic predisposition are well recognized and extensively studied, the role of sex in OA has often been overlooked, despite long‐standing evidence of sexual dimorphism in its prevalence, incidence, and severity [2, 3, 4, 5].

Like many other human diseases [6, 7, 8], OA exhibits sex‐specific distributions [2, 3, 4, 5]. Key features, such as prevalence, age of onset, progression, clinical prognosis, and response to treatment, differ between men and women. Notably, women are more susceptible to both the onset and progression of OA. The incidence of OA increases sharply in women, particularly after menopause around the age of 50, while this trend is not observed in men [9, 10]. As of 2019, more than 500 million people worldwide are affected by OA, with females representing over 60% of those cases [11]. In addition to a higher incidence and prevalence, women often experience faster symptom progression, greater functional disabilities, and poorer therapeutic outcomes compared with men, even when structural damage is similar [4]. Despite these well‐documented sex differences, most studies investigating OA risk factors and disease models have largely overlooked the impact of sex on the prevalence and clinical course of this disease.

Sex‐related disparities in OA are influenced by a variety of factors, including anatomical differences, mechanical loading patterns, hormone changes, and behavioral or socio‐cultural influences. At the tissue level, women tend to have thinner cartilage, a higher likelihood of excess body weight, increased joint instability, and more uneven mechanical loading than men. On the cellular level, preclinical studies have shown that chondrocytes from females display distinct behaviors, such as altered gene expression of inflammatory cytokines and sex hormone‐related transcriptional factors (TFs), as well as variations in responsiveness to hormone stimulation [2]. In addition to these chondrocyte‐specific differences, the composition of synovial fluid also differs between female and male OA patients. Pan et al. reported that women exhibit elevated levels of inflammatory cytokines, whereas men present with increased concentrations of catabolic enzymes, such as matrix metalloproteinases (MMPs), as well as higher levels of anabolic growth factors and sulfated glycosaminoglycans (sGAGs) [12]. Supporting these findings, Xue and colleagues demonstrated greater expression and production of pro‐inflammatory cytokines in synoviocytes derived from female rats compared with males [13]. As in many other tissues [14], sex hormone‐related TFs are believed to play a critical role in mediating sex‐specific responses in OA [15]. Among these, estrogen has been extensively investigated for its potentially protective effects in OA development and progression [16]. However, the efficacy of estrogen replacement therapy and selective estrogen receptor modulators in preserving and/or regenerating joint tissue in OA remains unclear and, in some cases, controversial [17, 18].

Despite accumulating evidence that sex is a critical biological variable in the pathophysiology of OA, the molecular mechanisms underlying sexual dimorphism in OA remain poorly understood. This knowledge gap is partly due to the limited number of studies specifically addressing sex differences at the molecular level. However, the growing availability of multi‐omics data nowadays offers an opportunity to investigate sex‐based differences in the human joint transcriptome that have not been previously explored. In our sex‐aware analysis of the bulk RNA‐seq dataset GSE114007 [19], we identified a set of highly correlated OA‐associated sex‐biased genes in human knee articular cartilage. These findings were validated across four independent datasets derived from diverse sources. Notably, these sex‐specific gene sets were enriched in broad yet distinct biological processes, underscoring the differential molecular pathways involved in male and female OA pathogenesis. To further investigate the regulatory basis of these transcriptomic differences, we integrated RNA‐seq findings with the ATAC‐seq dataset [20]. This analysis revealed that hormone‐related TFs are key regulators of sex‐biased gene expression, consistent with the known roles of sex hormones in joint biology. Together, our work provides new insights into sex‐specific transcriptomic signatures and regulatory mechanisms in human OA articular cartilage. These findings not only advance our understanding of sex differences in OA but also offer a foundation for developing sex‐based, targeted therapeutic strategies.

## 2 Results

### 2.1 Sex Effects on Gene Expression Exist in Normal Cartilage

**FIGURE 1:**
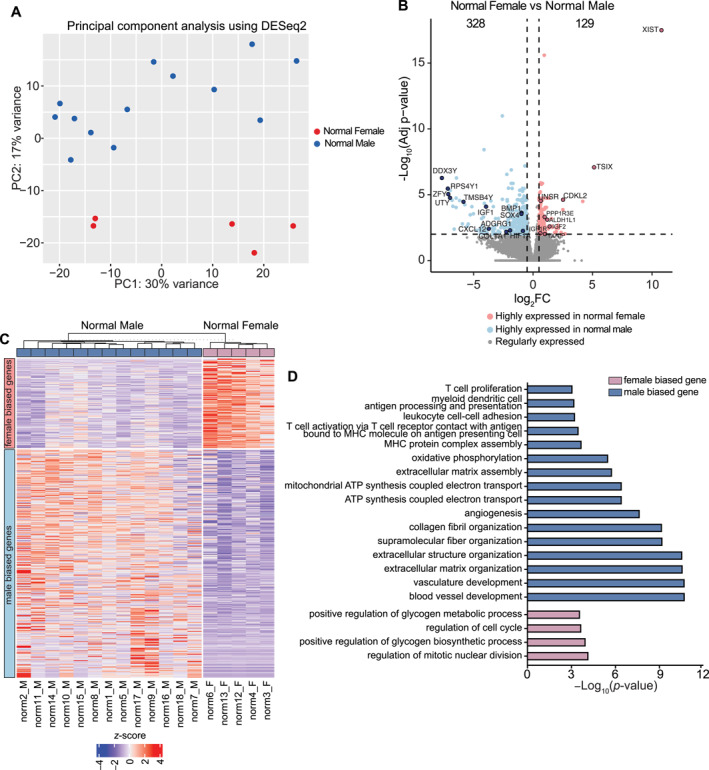
Transcriptomic analysis of normal cartilage revealed fundamental sex differences at the molecular level. (A) Principal component analysis of RNA‐seq data from normal cartilage of female and male patients. Female: red; male: blue. (B) Sex biased differentially expressed genes (DEGs) between normal female and male cartilage. Sex‐DEGs were identified using statistical cutoffs of log_2_FoldChange (log_2_FC) > 0.5 and false discovery rate (FDR) < 0.01. (C) Heatmap showing expression of female‐biased (129) and male‐biased (328) genes in normal female and male cartilage. (D) Enriched biological process among female‐biased and male‐biased gene sets.

To investigate the sex‐biased effects on the human chondrocyte transcriptome, we analyzed the bulk RNA‐seq dataset GSE114007 [19], which included quantified gene expression of both normal articular cartilage from 18 individuals (5 females and 13 males) and OA articular cartilage from 20 patients (12 females and 8 males) (Table 1). Principal component analysis revealed a global transcriptomic difference between normal female and male cartilage, demonstrating clear sex‐dependent separation (Figure 1A). To further explore the sex‐specific expression pattern in articular cartilage, we compared gene expression in normal female and male cartilage. We discovered a total of 457 sex‐specific differentially expressed genes (sex‐DEGs) with criteria of a log_2_FoldChange > 0.5 and padj < 0.01, including 26 genes on the *X* chromosome and 18 genes on the Y chromosome. Notably, several known OA‐associated genes were highlighted in the volcano plot (Figure 1B). Of these, 129 genes were highly expressed in females (female‐biased genes), and 328 genes were expressed at greater levels in males (male‐biased genes) (Figure 1B–C). These 457 sex‐DEGs represent 2.1% (457/21,435) of all tested genes in human chondrocyte transcriptome, consistent with previously reported ratios (1.3%–12.9%) of sex‐biased expression across 44 human tissue sources [21]. Furthermore, as noted in the original study [19], no significant differences were found in the general heathy status of donors, body mass index, or tissue sampling processes between the normal and OA samples. Thus, at physiologically homeostatic state, sex bias may exert a moderate influence on human chondrocyte transcriptome.

**TABLE 1:** Demographic information of patients listed in GSE114007.

Group	Sample size	Age range (yrs)	Mean age (yrs)	OA score range	Mean OA score	BMI
Normal female	5	27–57	42	1–1	1	32.4 ± 8.0
Normal male	13	18–61	34.5	1–1	1	
OA female	12	52–82	66.3	4–4	4	30.7 ± 8.1
OA male	8	51–71	64.9	4–4	4	

To gain insight into the cellular functions influenced by sex‐biased genes, we performed gene ontology enrichment analysis separately for female‐ and male‐biased gene sets. Notably, we observed distinct biological processes (FDR < 0.05) enriched with female‐ and male‐biased genes (Figure 1D). Female genes were particularly enriched in pathways related to cell cycle processes, glycogen biosynthesis, and energy reserve metabolic processes (Figure 1D). In contrast, the primary clusters driven by male genes included cellular matrix (ECM) organization, energy metabolism, oxidative respiration, blood vessel morphogenesis, and immune response (Figure 1D). Similarly, interaction network analysis using ClueGO [22] revealed distinct functionally grouped clusters enriched with female‐ and male‐biased genes (Figure S1). Together, these results indicate that sex‐biased genes are involved in a variety of biological functions and pathways, many of which are critical for regulating chondrocyte homeostasis and maintaining articular cartilage integrity. The distinct functional clusters specific to each sex may contribute to the etiology of OA with marked sex differences.

### 2.2 Sex Exhibits Comparatively Modest Effects Compared to the Influence of OA on Chondrocyte Transcriptome

**FIGURE 2:**
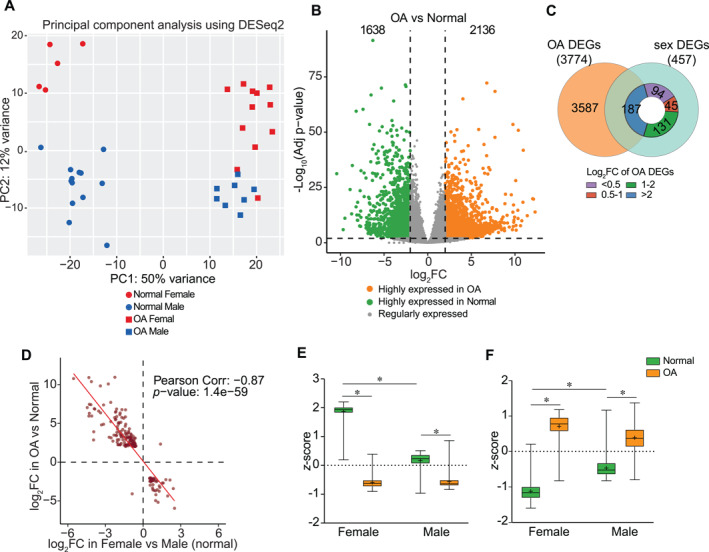
Shared DEGs between sex‐biased and OA‐specific subjects exhibit strong correlation. (A) Principal component analysis of RNA‐seq data from normal and OA cartilage of female and male patients. Dot: normal; square: OA; female: red; male: blue. (B) OA‐DEGs between normal and OA cartilage, irrespective of sex. OA‐DEGs were identified using statistical cutoffs of log_2_FC > 2 and FDR < 0.01. (C) Number of shared DEGs (sex‐OA‐DEGs) between sex‐biased and OA‐specific DEGs when varying statistical cutoffs for identifying OA‐DEGs. (D) Pearson's correlation between sex‐biased and OA‐related differentiated expression of sex‐OA‐DEGs. Expression (*z*‐score of RPKM) of the (E) female‐OA gene set (44 genes) and (F) male‐OA gene set (143 genes). *p‐value < 0.05. RPKM, reads per kilobase per million mapped reads.

Next, to assess the impact of sex‐biased genes on the regulation of OA, we included all samples from the original study in our analysis and compared the gene expression in arthritic cartilage to that in normal cartilage regardless of sex (20 OA samples and 18 normal samples). When both disease and sex were considered as biological variables, principal component analysis revealed a clear separation, with a more robust distinction between OA and normal cartilage (PC1, 50% of variance), along with a distinct sex‐dependent difference transcriptome within both normal and OA cartilage (PC2, 12% of variance) (Figure 2A). This suggests that sex exhibited comparatively modest effects compared with the influence of OA on gene expression in human chondrocyte transcriptome. In contrast to 457 sex‐DEGs identified, 3774 genes were differentially expressed (OA‐DEGs) in arthritic chondrocytes relative to the normal with criteria of a log_2_FoldChange > 2 and padj < 0.01. Among these genes, 2136 were significantly elevated in OA cartilage, and 1638 were highly expressed in normal cartilage (Figure 2B).

Among the 457 identified sex‐DEGs, 187 (40.9%) were significantly dysregulated in OA and classified as OA‐DEGs based on the criteria established earlier. Furthermore, another 176 of sex‐DEGs (38.5%) exhibited significant but more modest expression changes in OA cartilage compared with normal cartilage (0.5 < log_2_FoldChange < 2) (Figure 2C). The considerable sharing (*p* < 1.925e‐32, hypergeometric test) of sex‐DEGs with OA‐DEGs implies that sex may not only be a confounding variable but also a key determinant in OA pathogenesis. Of note, we observed a strong correlation (Pearson coefficient: −0.87, *p*‐value: 1.4e‐59) among these shared DEGs when differentiated by sex versus OA (Figure 2D). Specifically, of the 187 OA‐associated sex‐DEGs, 97.7% (43/44) of female‐biased genes were significantly decreased, while 99.3% (142/143) of male‐biased genes showed marked elevation in arthritic chondrocytes, implicating female and male gene sets likely have distinct impacts on OA (Figure 2C–D). Interestingly, when comparing OA with sex‐matched normal cartilage, both female and male gene set associated with OA (187 shared sex‐OA‐DEGs) demonstrated greater degree of dysregulation in response to OA development in female OA patients than in males (Figure 2E–F). This finding suggests that these sex‐OA‐DEGs in male cartilage are more resistant to molecular alterations during OA, whereas those in females are more susceptible to dysregulation of gene expression in response to OA.

**FIGURE 3:**
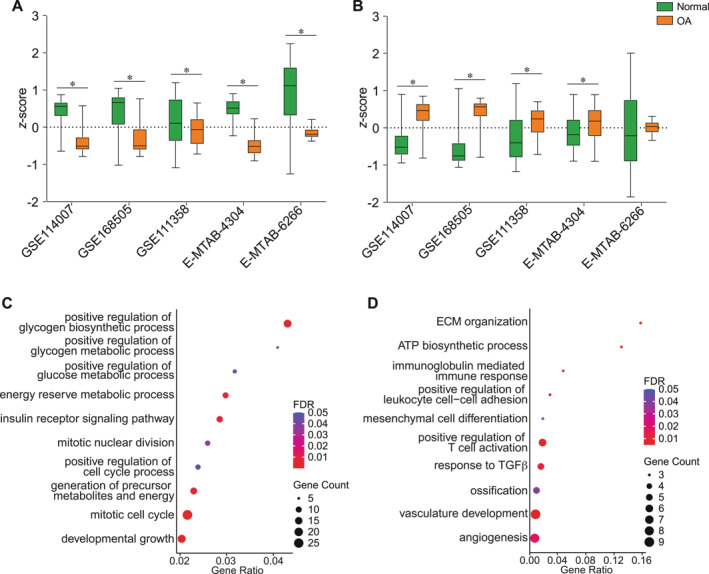
Validation of sex‐OA‐DEGs across independent datasets and gene oncology enrichment analysis. Expression (*z*‐score of RPKM) of the (A) 44 female‐OA genes and (B) 143 male‐OA genes across five independent datasets: GSE114007, GSE168505, GSE111358, E‐MTAB‐4304, and E‐MTAB‐6266. *p‐value < 0.05. Biological processes enriched in the (C) female‐OA gene set (44 genes) and (D) male‐OA gene set (143 genes).

To minimize potential bias from analyzing a single dataset and to provide additional support for the impact of sex‐specific OA‐DEGs on OA, we validated our identified gene sets across multiple diverse datasets from different sources. In addition to GSE114007, four eligible datasets, including GSE168505, GSE111358, E‐MTAB‐4304, and E‐MTAB‐6266, were selected for further analysis. Notably, the identified 44 female‐biased OA‐DEGs showed significantly decreased expression in OA cartilage compared with their corresponding controls across all five datasets (Figure 3A). In contrast, the identified male‐biased OA‐DEGs were significantly upregulated in OA cartilage across all datasets, except dataset E‐MTAB‐6266 (Figure 3B). This external validation with multiple, diverse datasets suggests that the female‐ and male‐biased gene sets have distinct impacts on OA, possibly acting as protective and risk genes for the disease, respectively.

### 2.3 Female‐Specific and Male‐Specific OA‐DEGs Are Involved in Distinct Biological Processes

Gene ontology enrichment analysis of the shared sex‐OA‐DEGs was performed separately for each sex‐specific gene set (female‐OA‐DEGs and male‐OA‐DEGs) to examine the biological processes affected (FDR < 0.05) (Figure 3C−D). Similar to the biological processes and functional clusters identified with sex‐biased genes (Figure 1D and Figure S1), female‐OA‐DEGs were primarily involved anabolic processes, such as cell cycle regulation, glucose metabolism, glycogen biosynthesis, and energy reserve metabolism (Figure 3C). Conversely, male‐OA‐DEGs were mainly associated with metabolically active and catabolic processes, such as oxidative phosphorylation, immune response, angiogenesis, and ECM turnover (Figure 3D). Given the overall change in the sex‐OA‐DEGs during OA (Figure 2E–F) and the distinct biological processes identified for each sex‐specific set, it is plausible to speculate that the female‐OA‐DEGs may have protective effects against cartilage degeneration, while male‐OA‐DEGs could adversely impact the maintenance of cartilage homeostasis. Future studies are warranted to corroborate this hypothesis, as the observed patterns may arise from a broad spectrum of molecular mechanisms.

### 2.4 Enrichment of Sex Hormone‐Related Transcriptional Factor Binding Motifs in Open Chromatin Regions (OCRs) of Sex‐OA‐DEGs

Transcriptional factors (TFs) are known to contribute to evolutionary changes in sex bias [23], and there have been recent studies of sex‐biased gene regulation by TFs across multiple human tissues [14, 21]. In the present study, to explore the regulatory relationship between the sex hormones‐controlled TFs and sex‐biased genes, we first assessed the gene expression of six known sex hormones‐related TFs, including androgen receptor (AR), estrogen receptors (ESR1 and ESR2), and Estrogen‐related receptors (ESRRA, ESRRB, and ESRRG). Estrogen‐related receptors (ESRRs), often referred to as orphan nuclear receptors, are structurally homologous to classical ERs and share substantial transcriptional targets with ESR1, but not ESR2 [24, 25], implying shared transcriptional networks driven by ESR1 and ESRRs. Although early studies demonstrated that estrogens do not bind to ESRRs and do not directly regulate their expression or transcriptional activity [26, 27], subsequent research has established crosstalk between ESRRs and ERs [28]. For instance, ESRRA can modulate ESR1‐mediated response of shared target genes in a 17β‐estradiol (E2)‐dependent manner, despite the absence of direct binding to E2 [29].

**FIGURE 4:**
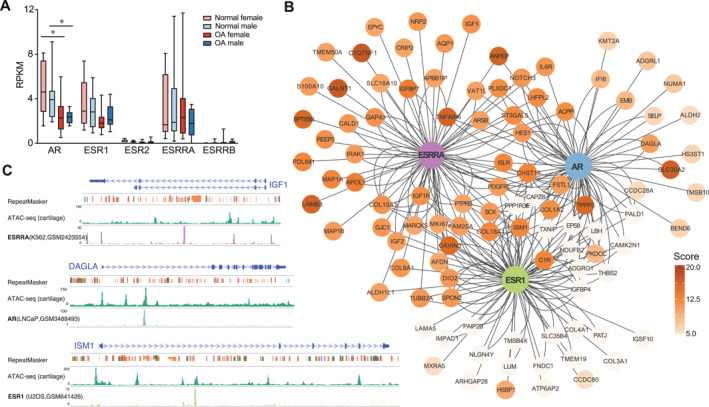
Sex‐OA‐DEGs are potentially regulated by sex hormone‐related TFs. (A) Expression profiles of sex hormone‐related TFs in normal and OA cartilage for each sex. (B) Motif enrichment for AR, ESR1, and ESRRA in open chromatin regions (OCRs) of sex‐OA‐DEGs. Color scale indicates the motif occurrence score. Central nodes (blue, green, and purple) represent TFs; surrounding genes connected by lines indicate potential target genes under regulation of each TF. (C) Examples of previously validated AR, ESR1, and ESRRA binding motifs in different types of cells overlapping with OCRs of DAGLA, ISM1, and IGF1 in human cartilage, respectively.

Analysis of the RNA‐seq dataset GSE114007 revealed that all five TFs, except for ESRRG, were detected in human chondrocytes (Figure 4A). In contrast to *ESR2* and *ESRRB* that were minimally expressed, *AR*, *ESR1* and *ESRRA* exhibited relatively moderate expression levels; thus, subsequent analysis focused on these three TFs. Additionally, both *AR* and *ESR1* were downregulated in OA cartilage, with *AR* showing a significant reduction in OA compared with their sex‐matched normal cartilage (Figure 4A), while expression of ESRRA showed no apparent changes. This suggests that the dominant sex hormone‐related TFs, AR and ESR1, may play a role in OA pathogenesis, potentially involving sex‐biased expression.

To test for enrichment of hormone‐related transcriptional factor binding sites (TFBSs) of the three TFs (AR, ESR1, and ESRRA) in open chromatin regions (OCRs) of human cartilage, we integrated our findings with an analysis of Assay for Transposase‐Accessible Chromatin with sequencing (ATAC‐seq) dataset GSE108301 with intact knee cartilage obtained from eight individuals (Table 2) [20]. To capture all OCRs relevant to the human chondrocyte genome, we combined all ATAC peaks and generated a comprehensive list of OCRs using the methods described previously [30]. We identified a total of 84,983 OCRs (each linked to its nearest gene with a maximum extension of 50 kb), among which 1230 were associated with 165 sex‐OA‐DEGs. Specifically, TFBSs of AR, ESR1, and ESRRA, were observed in OCRs of 44, 53, and 57 (a total of 101) sex‐OA‐DEGs, respectively (Table 3). Notably, candidates such as SCX, HES1 (Figure S2), and LBH, are of particular interest since they are TFs or TF‐cofactors (Figure 4B). Additionally, TFBSs of the three sex‐hormone receptors were found in several known genes involved in glucose and energy metabolism, including *IGF1* (Figure 4C), *IGF2*, *IGF1R*, and *ALDH2* (Figure 4B), indicating possible regulation of cellular metabolism mediated by sex hormones. Moreover, OCRs of other fibrotic markers, such as *COL3A1* and *COL1A2* (Figure S2), were enriched for sex hormone‐related TFBSs. Together, these results suggest that sex‐hormone related TFs regulate sex‐biased expression involved in various biological functions related to cartilage homeostasis and OA pathogenesis; however, they also indicate that additional TFs or molecular mechanisms play a role in sex‐biased expression as evidenced by the remaining 86 genes lacking sex hormone‐related TFBSs in their OCRs. Further studies utilizing multi‐omics approaches are needed to investigate the comprehensive genetic mechanisms underlying sex differences in OA prevalence and severity.

**TABLE 2:** Demographic information of patients listed in GSE108301.

Sample ID	Patient ID	KL‐grade	Age (yrs)	Gender	BMI	Left/Right
oLT_01	160908	4	75	M	29.2	R
oLT_02	160927	4	78	F	23.3	L
oLT_03	161004	3	86	F	25.3	R
oLT_04	161018	4	65	F	28.6	L
oLT_05	161027	4	70	F	23.5	R
oLT_06	161115	4	71	F	22.5	L
oLT_07	161124	4	78	F	19.8	R
oLT_08	161212	4	82	F	29.9	R

Abbreviation: oLT, outer region of lateral tibial plateau, representing intact cartilage.

**TABLE 3:** The number of sex‐OA‐DEGs potentially targeted by sex hormone‐related TFs, along with the TFBSs within the OCRs of these genes.

Sex‐OA‐DEG (187)	AR‐associated		ESR1‐associated		ESRRA‐associated	
		TFBS/sex‐OA‐DEG		TFBS/sex‐OA‐DEG		TFBS/sex‐OA‐DE
Gene	44	47.06%	53	57.22%	57	47.59%
Total OCR	54		73		85	
Total TFBS	88		107		89	

### 2.5 Transposable Elements Are Involved in Cartilage Biological Processes and OA

**FIGURE 5:**
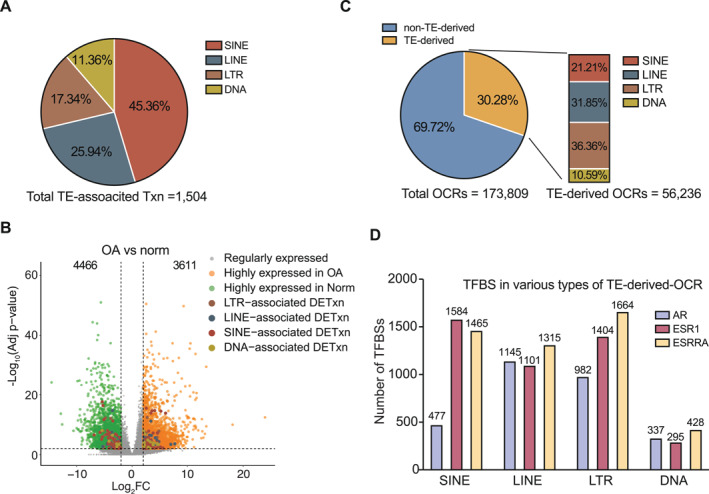
Transposable elements (TEs) are implicated in cartilage biology and OA. (A) Proportions of different TE classes among the 1504 TE‐derived transcripts in human cartilage. (B) Differentially expressed transcripts (DETxns) between normal and OA cartilage regardless of sex. OA‐DETxns were identified using statistical cutoffs of log_2_FC > 2 and FDR < 0.01. TE‐derived OA‐DETxns are highlighted. (C) Proportion of TE‐derived OCRs among all 173,809 OCRs in human cartilage, and the distribution of TE classes among TE‐derived OCRs. (D) Enrichment of AR, ESR1, and ESRRA TFBSs across different TE‐derived OCR types.

Transposable elements (TEs) are repetitive genomic sequences that comprise approximately 45% of the human genome. To investigate the functional role of TEs in human cartilage and their potential contribution to the pathogenesis of OA, we first examined transcripts initiated from TE‐derived transcription start sites (TSSs). We identified 1504 transcripts in human cartilage that originated from TE‐derived TSSs, with nearly half derived from SINE elements (Figure 5A). Subsequent differential expression analysis revealed that 224 of these TE‐derived transcripts were significantly altered during OA onset and progression (Figure 5B). Among them, 120 transcripts of 118 genes were upregulated in OA samples. Conversely, another 104 TE‐derived transcripts of 103 genes were downregulated in OA and were enriched in protein folding and metabolic processes (Figure S3).

TEs harbor numerous TFBSs and have been co‐opted as *cis*‐regulatory elements (CREs) to fine‐tune gene regulatory networks (GRNs) during human evolution. In human cartilage, we identified 56,236 OCRs that are TE‐derived, representing approximately 30% of the total OCRs in this tissue (Figure 5C). TE classification analysis revealed that LINE and LTR elements are the predominant contributors to these regulatory regions, consistent with observations in other human tissues. Motif enrichment analysis further demonstrated that TE‐derived OCRs are enriched for sex hormone receptor binding sites. Specifically, we found that 2941 TE‐OCRs contain AR motifs, 4384 contain ESR1 motifs, and 4872 contain ESRRA motifs (Figure 5D). Interestingly, SINE and LTR elements showed a strong preference for harboring estrogen receptor (ESR1/ESRRA) binding sites. This is consistent with prior findings in human MCF‐7 breast cancer cells, where SINE elements were closely associated with estrogen response [31]. These results suggest a potential hormone‐responsive regulatory role of SINE‐derived OCRs in human cartilage and implicate hormone‐mediated regulation in the development and progression of OA.

## 3 Discussion

Through a sex‐aware transcriptomic analysis of normal human knee articular cartilage, we identified a set of highly correlated, OA‐associated, sex‐biased genes that are enriched in a broad range of biological processes. Integrating this RNA‐seq data with an ATAC‐seq dataset from intact human articular cartilage, we found that sex hormone‐related TFs regulate the expression of these sex‐biased genes, as anticipated. Our work characterizes sex differences in the human knee joint transcriptome within the context of OA and highlights the potential genetic regulatory mechanisms underlying these differences. These insights enhance the biological interpretation of sexual dimorphism in OA and lay the groundwork for identifying sex‐specific biomarkers and developing targeted therapies that address the unique needs of both female and male OA patients.

Given previous findings that sex effects on gene expression are widespread yet generally modest across human tissues [21], we applied a less stringent statistic threshold to identify sex‐biased genes (log_2_FoldChange > 0.5), compared to the more rigorous cutoff used for OA‐specific genes (log_2_FoldChange > 2). Consistent with prior observations, we found that sex exerts a measurable but smaller influence on gene expression in human articular cartilage relative to the more pronounced molecular changes driven by OA. Specifically, our analysis identified 457 sex‐biased genes (median fold change = 1.82) and 3774 OA‐specific genes (median fold change = 8.75), corresponding to 2.1% (457/21,435) and 17.6% (3774/21,435) of all tested genes in human articular chondrocyte transcriptome, respectively. Despite the relatively modest number of sex‐biased genes, we observed a significant overlap between sex‐biased and OA‐specific gene sets, suggesting that sex is not merely a confounding factor but a key biological determinant in OA pathogenesis. This conclusion is further supported by a highly inverse correlation (coefficient: −0.87) in the expression patterns of sex‐biased genes following OA induction. Notably, 43 of 44 female‐biased genes were significantly downregulated in arthritic cartilage, whereas 142 of 143 male‐biased genes were significantly upregulated, highlighting a sex‐dependent molecular response to OA.

Our GO enrichment analysis revealed distinct biological processes associated with the two sets of sex‐biased genes. Female‐biased genes were primarily enriched in anabolic pathways, including glycogen biosynthesis, energy reserve metabolism, and cell cycle regulation. In contrast, male‐biased genes were predominantly linked to catabolic processes such as oxidative phosphorylation, energy metabolism, ECM turnover, vessel development, and immune response. These trends were consistent with the enrichment profiles of sex‐biased OA‐responsive genes. Further supporting these findings, we observed that female‐specific OA‐responsive genes were generally downregulated, whereas male‐specific OA‐responsive genes were unregulated. This pattern, along with their respective functional annotations, suggests that, under physiological homeostasis, female‐specific OA‐responsive genes may play a protective role against OA, whereas male‐specific OA‐responsive genes may contribute to cartilage degradation and disease exacerbation. This interpretation is aligned with observations from post‐traumatic OA mouse models, where male mice tend to develop a more severe OA than females following joint injury [32, 33, 34, 35]. However, these preclinical findings contrast with epidemiological data in humans, which consistently show higher OA prevalence and severity in women [5, 9, 15, 36, 37, 38]. The reasons for this discrepancy remain unclear but may reflect differences in experimental models or age mismatch, as most preclinical studies utilize young mice that do not fully capture the complexity of human OA, particularly age‐related degeneration.

Therefore, high‐quality, prospective preclinical and clinical studies are essential to elucidate sex‐associated factors contributing to OA development and progression. Such studies will be critical for advancing our understanding of sex differences in OA and for informing the development of sex‐specific diagnostic tools and therapeutic strategies. Nonetheless, our analysis suggests that the sex‐specific OA‐responsive genes are more susceptible to be dysregulation in female cartilage than in male cartilage. This molecular vulnerability may help explain why female patients often experience more severe OA‐related symptoms than males [5, 36, 37, 38]. In support of this concept, Li et al. utilized the same dataset and reported that female patients exhibit greater impairment of extracellular matrix turnover, a key process in cartilage degeneration. At the transcriptomic level, OA onset and progression in females and males involve distinct sets of gene alterations, although the mechanisms driving these sex‐specific changes remain to be fully elucidated [39].

In efforts to understand the molecular basis of sex differences in OA, it is important to recognize that the relationship between transcriptomic patterns and complex phenotypic outcomes is likely confounded by various compensatory and buffering mechanisms, as is common in many diseases and traits [40]. Regardless of detectable sex differences at the transcriptomic level, many biological characteristics at the phenotypic level are shared between females and males. Notably, the sex differences observed in this study are based on a snapshot of a relatively young cohort of normal cartilage donors (mean age of 36.6) compared to the OA group (mean age of 65.7). As such, transcriptomic changes that emerge later in life, particularly those relevant to OA, may not be fully captured. Additionally, the imbalance in sex representation among the normal cartilage donors (5 females and 13 males) limits the statistical power to detect sex‐biased genes, contributing to the identification of 129 female‐biased and 328 male‐biased genes in the current cohort. These limitations underscore the need for larger, sex‐balanced, and age‐matched cohorts that include OA populations to more accurately characterize transcriptomic sex differences. Furthermore, sex is inherently intertwined with lifestyle factors and environmental exposures [41]. For instance, sex differences in the association between smoking and knee OA have been documented [42], emphasizing the challenge of disentangling biologically intrinsic sex differences from those shaped by gendered environments. Addressing this complexity remains a key objective for future research.

Our analysis of the genetic regulation of sex‐biased genes suggests that sex hormones may play a critical role in the observed transcriptomic patterns. An early landmark study by Ma et al. demonstrated that female mice subjected to destabilization of the medial meniscus (DMM) developed more severe OA following ovariectomy (OVX), indicating the protective effects of ovarian hormones [43]. This protective effect has been corroborated in OVX rat models [44, 45], where estrogen deficiency accelerated cartilage degradation and bone turnover, which could be mitigated by supplementation of estrogen [45] or treatment with estrogen receptor modulator [44, 45]. More recently, Glimer et al. showed that chemically induced menopause in middle‐aged female mice exacerbated cartilage degeneration likely due to loss of 17b‐estradiol and progesterone [46]. While preclinical research consistently supports the beneficial effects of estrogen in preserving joint integrity, findings from large‐scale observational studies and clinical trials in humans have been mixed. In particular, the impact of estrogen replacement therapy on OA risk and progression in postmenopausal women remains controversial [17, 47, 48]. These discrepancies highlight the need for further mechanistic studies in both animal models and human cohorts to better understand the context‐dependent effects of sex hormones on joint health and to inform therapeutic strategies tailored to sex and hormonal status.

The role of male sex hormones in osteoarthritis (OA) appears complex and context‐dependent, suggesting potential interactions with other risk factors in disease initiation and progression. In orchiectomized (ORX) mice, OA severity following destabilization of the medial meniscus (DMM) was reduced, and administration of dihydrotestosterone (DHT) restored OA severity to levels seen in intact males, indicating that androgens may exacerbate OA susceptibility in males [43]. However, contrasting evidence from a rat model of monoiodoacetate‐induced OA showed that ORX worsened disease symptoms, implying a protective role for testosterone under certain conditions [49]. Human studies have similarly produced mixed findings: Freystaetter et al. reported that higher serum testosterone levels were associated with reduced knee pain in both men and women following total knee replacement [50], while Cheng et al. found that low serum testosterone significantly increased the risk of developing arthritis [51]. These observations highlight that the effects of male sex hormones on joint health are influenced by additional factors, such as the type of OA model, injury patterns, systemic metabolic status, and inflammation, all of which may modulate androgen actions in cartilage and subchondral bone. Further mechanistic studies are needed to clarify how sex‐related hormonal pathways intersect with other risk factors to drive the onset and progression of OA.

Moreover, when discussing sex hormones and their contributions to sex‐related differences in OA, it is important to emphasize several relevant factors, including serum levels of sex hormones, local production, and the presence and functionality of receptors and associated signaling pathways [15]. Although the gene expression and/or protein levels of these receptors have been evaluated in many studies, functional assays are often lacking. Further investigations, complemented by functional tests, are warranted to fully understand the impact of sex hormones and their TFs on sex differences in OA.

Our findings on sex‐biased gene expression in cartilage support the concept that understanding sex differences in OA requires integrating the fundamental impact of sex on diverse biological processes involved in joint homeostasis. This includes not only systemic factors but also cell type–specific regulatory mechanisms within the joint, both in healthy and diseased states. In addition to transcriptomic differences, our analysis suggests that sex‐biased genetic regulation may contribute to the observed sexual dimorphism in OA. Importantly, the regulation of sex‐biased genes in cartilage likely extends beyond canonical sex hormone signaling. Our results point to the presence of additional sex‐specific genetic architectures that may underlie distinct cartilage physiology in males and females. These findings call for future experimental and functional studies to validate the observed patterns and to further elucidate the genetic and epigenetic mechanisms governing sex‐biased gene expression, thereby clarifying their contribution to OA pathogenesis.

## 4 Methods

### 4.1 RNA‐Seq Data Processing

The published bulk RNA sequencing (bulk RNA‐seq) dataset GSE114007 of normal (5 females, age 27–57 with a mean age of 42; 13 males, age 18–61 with a mean age of 34.5) and arthritic (12 females, age 52–82 with a mean age of 66.3; 8 males, age 51–71 with a mean age of 64.9) knee articular cartilage was analyzed to identify sex differences in gene expression levels and their association with OA. According to the original study, there were no significant differences in the general health status of donors, body mass index, or tissue sampling processes between the normal and OA samples [19]. A total of 38 raw RNA‐seq FASTQ files and SRA data for this dataset were downloaded from NCBI GEO https://www.ncbi.nlm.nih.gov/geo/query/acc.cgi?acc=GSE114007.

Differential gene analysis was performed as follows: comparing 5 normal females with 13 normal males to identify sex‐biased gene expression in normal cartilage and comparing 20 OA cartilage samples to 18 normal cartilage samples (regardless of sex) to assess molecular changes at the transcriptome level in response to OA. As previously described, RNA‐seq data were processed using Cutadapt (v2.7; —quality‐cutoff = 15, 10 —minimum‐length = 36), FastQC (v0.11.4) and STAR (v2.5.2b; —quantMode TranscriptomeSAM —outWig‐Type bedGraph —outWigNorm RPM) for trimming, QC report, and mapping to the human genome (hg38) [52]. The QC reports of mapping results are shown in Table S1. Then, gene expression in the cartilage was quantified using featureCounts (‐p ‐T 4 ‐Q 10) based on UCSC gene annotation of the human genome [53].

### 4.2 Sex‐Biased Genes in Normal Cartilage and OA‐Induced Differentially Expressed Genes

RNA‐seq data were then normalized using the RUVSeq package, as described in a previous study [54]. Specifically, gene counts were first upper‐quantile normalized using edgeR [55], followed by factor analysis with residual calculations from the RUVr function in the RUVSeq package. After normalization, DESeq2 [56] was employed to identify sex‐biased genes that were significantly highly expressed in one sex compared to the other in normal cartilage The cutoff used for significance included a log_2_FoldChange > 0.5 and adjusted *p*‐value (padj) < 0.01. As for the comparison between OA and normal cartilage, both differentially expressed genes (DEGs) and differentially expressed transcripts (DETxns) were identified with a log_2_FoldChange > 2 and padj < 0.01, ensuring both specificity and sensitivity. The Venn plot of intersection for sex‐biased DEGs and OA‐specific DEGs was generated by jvenn [57].

The interactive network analysis of sex‐biased genes was performed using STRING [58] database showing the potential interactions and subsequently analyzed by ClueGO [22] in Cytoscape [59] to identify the functional enrichment of biological processes in identified network clusters.

### 4.3 Literature Search and Assessment of Selected Studies

To validate the sex‐OA‐DEGs identified in this work across different studies, a systematic search was conducted by consulting the NCBI GEO and EMBL‐EBI databases for high‐throughput sequencing datasets generated from studies published between 2004 and 2024. The following combinations of keywords were employed: “human”, “cartilage”, AND “osteoarthritis”. We included studies that met the following the criteria: human articular cartilage was individually sequenced, and both normal (or preserved) and osteoarthritic (or damaged ) samples were investigated. Exclusion criteria included articles that did not involve human cartilage, polled cartilage, or cells, in vitro culture of isolated chondrocytes, the presence of in vitro or ex vivo treatment, and genetic modifications used. Datasets were downloaded and processed as described earlier. After a quality control check, five datasets, including GSE114007, GSE168505, GSE111358, E‐MTAB‐4304, and E‐MTAB‐6266, were selected for further analysis. Mapping QC summaries for each independent dataset are shown in Table S1.

### 4.4 ATAC‐Seq Data Processing

Published Assay for Transposase‐Accessible Chromatin with sequencing (ATAC‐seq) dataset GSE108301 of 8 intact human knee cartilage (from 7 females and 1 male, age 65–86 with a mean age of 75.6) was analyzed to test for enrichment of hormone related transcriptional factor binding sites (TFBSs). A total of 8 raw ATAC‐seq FASTQ files and SRA data for this dataset were downloaded from NCBI GEO https://www.ncbi.nlm.nih.gov/geo/query/acc.cgi?acc=GSE108301.

ATAC‐seq data of eight intact cartilages were separately processed by the AIAP package that contained optimized ATA‐seq data and an analysis pipeline with default parameters [60]. The list of consensus open chromatin regions (OCRs) of intact human cartilage was built from ATAC peak counts from eight individuals using the methods described previously with modifications [30].

### 4.5 Motif Finding of Hormonal Receptors in OCRs and Gene Regulation Network

The OCRs were used to identify associated genes in intact human carriage with GREAT (version 4.0.4) [61]. The analysis settings of GREAT included the following: (1) Species assembly: Human, GRCh38; (2) Background regions: whole‐genome; (3) Association rule settings: Basal plus extension. The FIMO [62] software was used to scan TFBS motifs for AR, ESR1 and ESRRA in those OCRs based on the motif weigh matrix file (JASPAR_CORE_2023_vertebrates.‐meme) from JASPAR [63]. The OCRs associated genes containing sex hormone TFBSs were extracted to build the gene regulation networks and subsequently visualized by Cytoscape [59]. Examples of previously validated AR, ESR1, and ESRRA binding motifs in different types of cells overlapping with OCRs of genes in human cartilage were visualized by the WashU Epigenome Browser.

### 4.6 Identification of TE‐Derived Transcripts and TE‐Derived OCRs

The intersectBed tool was used to determine the number of transposable element (TE)‐derived transcripts and TE‐derived OCRs in human knee cartilage. TE types, including SINE, LINE, LTR, and DNA elements, were defined using the UCSC TE annotation for the human genome (hg38). Among the 55,705 transcripts expressed in human cartilage, TE‐derived transcripts were identified by overlapping their transcription starts sites (TSS) with annotated human TE regions using intersectBed. Similarly, TE‐derived OCRs were determined by overlapping the previously identified 173,809 OCRs (regardless of gene association) in human cartilage with TE regions, requiring at least a 25% overlap with each OCR.

## 5 Statistics

All computational analyses were performed using the R package (version 4.2.2), DESeq2 was used to identify the DEGs and DETxns in each condition as indicated, and a master list of OCRs of intact human cartilage was generated using the methods as previously reported [30]. The correlation of fold changes of sex‐biased genes and OA‐induced DEGs was examined by cor function in R with the Pearson method. The ToppGene suite [64] was employed to perform GO‐term enrichment analysis. All the details are provided in each Methods section.

Stringent statistical cutoffs were applied to identify sex‐specific DEGs (log_2_FoldChange > 0.5 and padj < 0.01), and OA‐specific DEGs and OA‐specific DETxns (log_2_FoldChange > 2 and padj < 0.01). Multiple comparisons were corrected and controlled by using the FDR method.

## Author Contributions

C.W. and T.L. performed sequencing data analyses. B.Z. guided the sequencing data analyses. M.S., S.L., L.F., and M.X. participated in data discussion and interpretation. C.W., B.Z., and J.S. participated in manuscript writing and revision. J.S. planned the general outline of the project, and finalized the manuscript.

## Ethics Statement

The authors have nothing to report.

## Conflicts of Interest

The authors declare no conflicts of interest.

## Supporting information


Supporting Information S1

**Table S1**: Mapping quality control in RNA‐seq data sets.

## Data Availability

The RNA‐seq datasets are available in the Gene Expression Omnibus (GEO) and ArrayExpress databases under the accession numbers GSE114007, GSE168505, GSE111358, E‐MTAB‐4304, and E‐MTAB‐6266. The ATAC‐seq dataset is accessible via GEO under accession number GSE108301.
