# Supplementary material for: Contribution of Sex‐Biased Expressed Genes in Osteoarthritis
Source: Smart Med. 2025 Oct 6;4(4):e70019. doi: 10.1002/smmd.70019 (PMC12503069; doi:10.1002/smmd.70019)
Supplement: Supplementary file 1 — Supporting Information S1 [file SMMD-4-e70019-s002.pdf]

Suppl. Figure 1. Functional enrichment of sex-biased genes in normal human articular cartilage. Interaction network of functional grouped clusters enriched by female-biased genes (**A**) and male-biased genes (**B**) were constructed using STRING databased and visualized by ClueGO. Different colors of nodes represent various Gene Ontology (GO) categories. Size of nodes reflect the significance of the GO terms. Edges between nodes indicate the protein-protein interactions of functional associations of enriched GO terms.

Suppl. Figure 2. Additional examples of previously validated AR, ESR1, and ESRRA binding motifs in different types of cells overlapping with OCRs of COL1A2 and HES1 in human cartilage.

Suppl. Figure 3. Gene ontology analysis of 104 downregulated TE-derived transcripts, corresponding to 103 genes, revealed enrichment in protein folding and metabolic processes.

Table S1. Mapping quality control in RNA-seq data sets

Suppl. Fig. 1

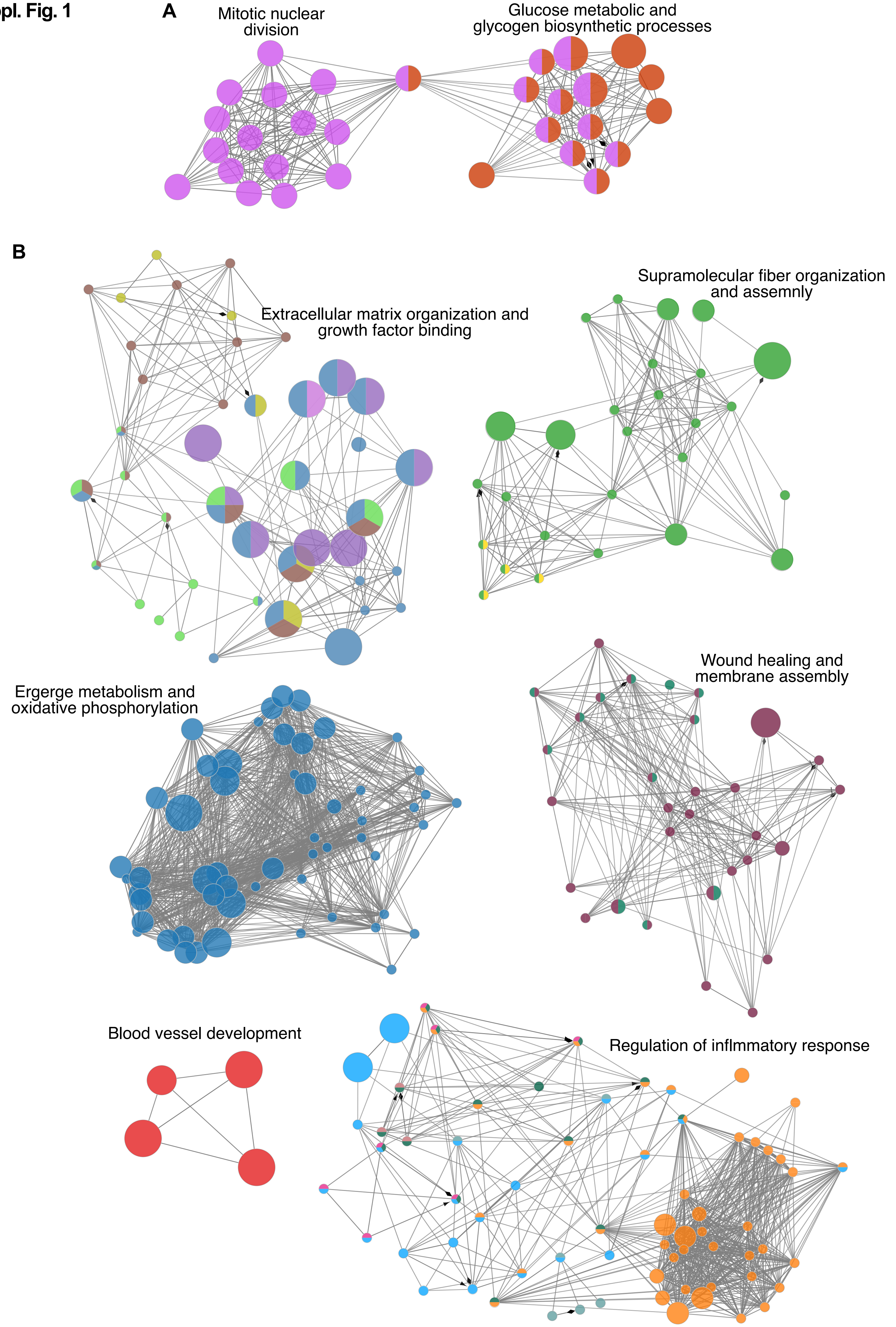
A
Mitotic nuclear division
Glucose metabolic and glycogen biosynthetic processes
B
Extracellular matrix organization and growth factor binding
Supramolecular fiber organization and assemnly
Ergerge metabolism and oxidative phosphorylation
Wound healing and membrane assembly
Blood vessel development
Regulation of inflmmatory response

Suppl. Fig. 2

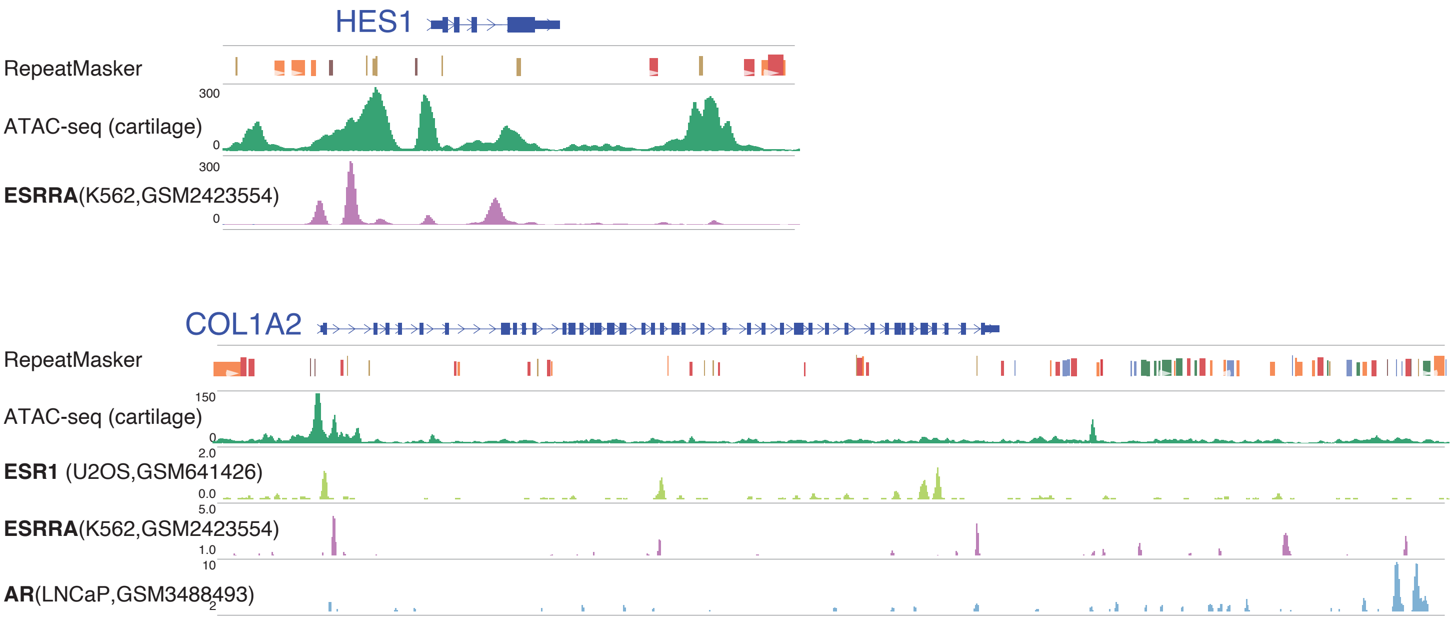

HES1
RepeatMasker
ATAC-seq (cartilage)
300
0
ESRRA(K562,GSM2423554)
300
0
COL1A2
RepeatMasker
ATAC-seq (cartilage)
150
0
ESR1 (U2OS,GSM641426)
2.0
0.0
ESRRA(K562,GSM2423554)
5.0
1.0
AR(LNCaP,GSM3488493)
10
2

Suppl. Fig. 3

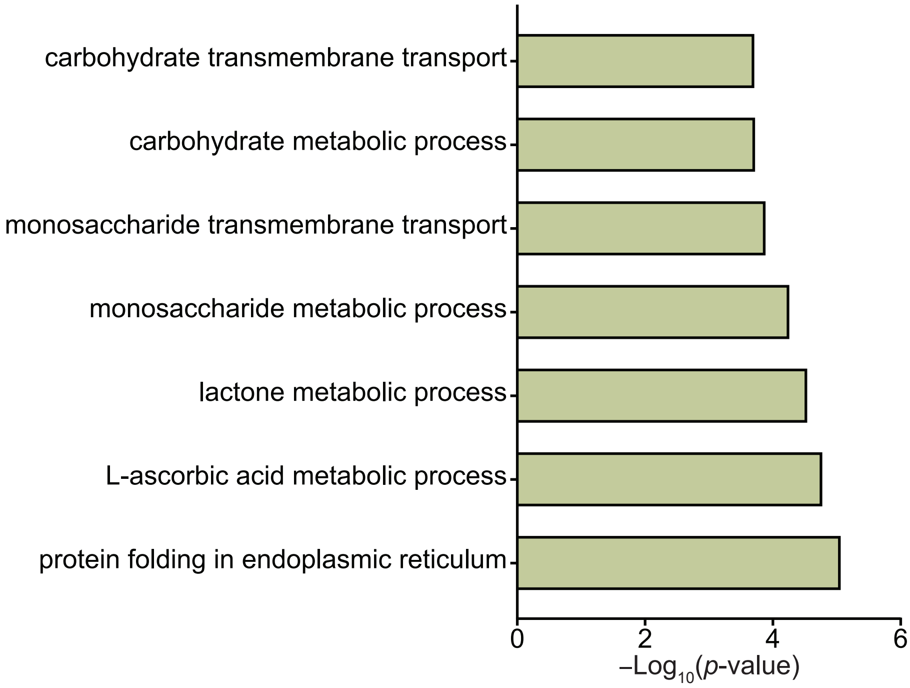
carbohydrate transmembrane transport
carbohydrate metabolic process
monosaccharide transmembrane transport
monosaccharide metabolic process
lactone metabolic process
L-ascorbic acid metabolic process
protein folding in endoplasmic reticulum
0
2
4
6
–Log10(p-value)
